# Similar Patterns of Dysautonomia in Myalgic Encephalomyelitis/Chronic Fatigue and Post-COVID-19 Syndromes

**DOI:** 10.3390/pathophysiology31010001

**Published:** 2024-01-05

**Authors:** Varvara A. Ryabkova, Artemiy V. Rubinskiy, Valeriy N. Marchenko, Vasiliy I. Trofimov, Leonid P. Churilov

**Affiliations:** 1 Laboratory of the Mosaic of Autoimmunity, Saint Petersburg State University, 199034 Saint-Petersburg, Russia; elpach@mail.ru; 2 Department of Pathology, Saint Petersburg State University, 199034 Saint-Petersburg, Russia; 3 Department of Hospital Therapy Named after Academician M.V. Chernorutskii, Research Institute of Rheumatology and Allergology, Pavlov First Saint Petersburg State Medical University, 197022 Saint-Petersburg, Russia; marchvn@mail.ru (V.N.M.); trofvi@mail.ru (V.I.T.); 4 Department of Medical Rehabilitation and Adaptive Physical Culture, Pavlov First Saint Petersburg State Medical University, 197022 Saint-Petersburg, Russia; rubinskiyav@1spbgmu.ru; 5 Laboratory of Microangiopathic Mechanisms of Atherogenesis, Saint Petersburg State University, 199034 Saint-Petersburg, Russia; 6 Saint Petersburg Research Institute of Phthisiopulmonology, 191036 Saint Petersburg, Russia

**Keywords:** chronic fatigue syndrome, COVID-19, post-COVID, heart rate variability, autonomic dysfunction

## Abstract

Background: There is a considerable overlap between the clinical presentation of post-COVID-19 condition (PCC) and myalgic encephalomyelitis/chronic fatigue syndrome (ME/CFS). Many of their common symptoms can be linked to dysregulation of the autonomic nervous system (dysautonomia). This study aimed to objectively assess autonomic function in a general group of patients with PCC and in a group of patients with ME/CFS whose disease was not related to COVID-19. We hypothesize that the similarity in the chronic symptoms of patients with PCC and ME/CFS extends to objective autonomic nervous system abnormalities. Methods: Synchronous recordings of an electrocardiogram and continuous dynamics of blood pressure in the digital artery using the Penaz method were obtained using the spiroarteriocardiorhythmography method in 34 patients diagnosed with ME/CFS, in whom the onset of the disease was not associated with COVID-19, 29 patients meeting the PCC definition and 32 healthy controls. Heart rate variability (HRV) and systolic and diastolic blood pressure variability (BPV) were assessed at rest and in tests with fixed respiratory rates. Indicators of baroreflex regulation (baroreflex effectiveness index and baroreflex sensitivity) were additionally determined at rest. Results: The total power and power of low-frequency and high-frequency of RR interval variability at rest as well as baroreflex sensitivity were significantly lower both in PCC and ME/CFS patients compared to healthy controls. Several diagnostic prediction models for ME/CFS were developed based on HRV parameters. During slow breathing, the HRV parameters returned to normal in PCC but not in ME/CFS patients. The correlation analysis revealed a close relationship of HRV, BPV parameters and baroreflex sensitivity with fatigue, but not with HADS depressive/anxiety symptoms in the ME/CFS and PCC patients. Conclusions: A similar pattern of HRV and baroreflex failure with signs of a pathological acceleration of age-dependent dysautonomia was identified in the ME/CFS and PCC patients. The clinical, diagnostic and therapeutic implications of these findings are discussed, in light of previously described relationships between inflammation, vascular pathology, atherosclerotic cardiovascular disease and autonomic dysfunction.

## 1. Introduction

ME/CFS is classified as a chronic neurologic disease and there is a vast collection of biomedical findings regarding this condition, although they are not well known to researchers and clinicians in other fields [1]. The disease in 49–93% of cases is preceded by infection [2]. There has been increased interest in ME/CFS recently because of its significant overlap with post-COVID-19 condition (PCC, also known as long COVID/post-COVID syndrome), with several studies estimating that 58% of patients with PCC fulfill the ME/CFS criteria [3]. Regarding pathophysiological mechanisms, evidence of neuroinflammation and objective abnormalities of orthostatic intolerance in PCC are very similar to those in ME/CFS patients [4,5]. These findings suggest that a subgroup of PCC and ME/CFS are probably the same disease which can be caused by different triggers. PCC was officially recognized by WHO in October 2021, and a clinical case definition was developed in a Delphi process. According to this definition, “post-COVID-19 condition occurs in individuals with a history of probable or confirmed SARS-CoV-2 infection, usually 3 months from the onset, with symptoms that last for at least 2 months and cannot be explained by an alternative diagnosis. Common symptoms include, but are not limited to, fatigue, shortness of breath, and cognitive dysfunction, and generally have an impact on everyday functioning. Symptoms might be new onset following initial recovery from an acute COVID-19 episode or persist from the initial illness. Symptoms might also fluctuate or relapse over time” [6]. There is evidence for a close relationship between ME/CFS and PCC regarding not only the symptomatology, but also the key mechanisms (neuroinflammation, dysautonomia and vascular pathology). In particular, as summarized by Komaroff and Lipkin [7], autonomic dysfunction has been well documented in both ME/CFS and PCC. Significant platelet hyperactivity and endothelial dysfunction as well as circulatory disorders were documented in both PCC and ME/CFS, although in PCC, possible pro-atherogenic evidence was registered [8], while in ME/CFS (also related to viral etiology), the circulatory disorders look like non-atherosclerotic cardiological disease and “it seems that neurological (autonomic) dysfunction underlies these abnormalities” [9]. Abnormalities of both the sympathetic and parasympathetic arms of the autonomic nervous system have been reported, with the imbalance favoring expression of the sympathetic system [7]. The concept that sinus node pacemaker activity and vascular resistance are under control of the autonomic nervous system has promoted the use of RR intervals and beat-to-beat arterial blood pressure measurements to evaluate the autonomic modulation of cardiovascular function. Among the proposed indices, heart rate variability (HRV), arterial blood pressure variability and baroreflex sensitivity (BRS) are the most accepted and well-known ones [10]. The responses of these parameters to deep breathing at different rates are the simplest tests to assess the activity of the parasympathetic and sympathetic nervous systems.

Our study aimed to objectively assess autonomic function in a general group of patients with PCC and in a group of patients with ME/CFS whose disease was not related to COVID-19. We hypothesized that the similarity in the chronic symptoms of patients with PCC and ME/CFS extends to objective autonomic nervous system abnormalities.

## 2. Materials and Methods

### 2.1. Participants

In this study, three group of subjects were involved (34 patients with ME/CFS that was not related to COVID-19; 29 patients with PCC; 32 healthy controls (HCs)). Patients with ME/CFS were included in the study if they met all three of the most commonly used sets of ME/CFS diagnostic criteria (Fukuda et al. (1994) CFS criteria [11], the Canadian Consensus criteria of ME/CFS (2003) [12] and the US Institute of Medicine, now called the National Academy of Medicine (IOM/NAM), criteria (2015) [13]). The details of the diagnostic processes can be found in the literature [14]. The diagnosis of PCC was defined based on the WHO criteria [6]. Patients with PCC were also evaluated for compliance to the Canadian Consensus criteria for ME/CFS (2003) and IOM/NAM criteria (2015). Age- and sex-matched HCs who did not have chronic fatigue were recruited through word-of-mouth from the local community. The common exclusion criteria for all groups were as follows: age <18 or >60 years; cardiac arrhythmias; structural heart diseases; current usage of beta-blockers; any acute illness during the last month before the study. The study was approved by the Biomedical Ethics Committee of I.P. Pavlov First Saint Petersburg State Medical University. All participants gave informed consent.

### 2.2. Measures

#### 2.2.1. Symptom Assessment Tools

The Multidimensional Fatigue Inventory (MFI) and Hospital Anxiety and Depression Scale (HADS) were used to assess symptoms in all participants. The MFI is a 20-item self-reported questionnaire which assesses general physical fatigue, reduced activity, reduced motivation and mental fatigue [15]. HADS represents a reliable scale for identifying and assessing the symptom severity in depression and anxiety disorders, both in patients with somatic diseases and in patients with endogenous mental disorders [16]. A score of 0–7 for each subscale (depression and anxiety symptoms) indicates “normal results”; 8–10 indicates “borderline results, or doubtful case of anxiety/depression”; and 11 or more indicates “probable case of anxiety/depression”.

#### 2.2.2. Assessment of Autonomic Nervous System Function

An integrated method of studying the cardiorespiratory system—spiroarteriocardiorhythmography (SACR) (“Intox”, Russia)—was applied to analyze heart rate variability (HRV), arterial blood pressure variability (BPV) and respiratory patterns. This device was recommended for use in medical practice by the Ministry of Health of Russia (registration certificate No 29/03020703/5869-04) and combines three physiological methods into an integrated hardware complex.

R-R interval data were recorded using a three-lead electrocardiogram (Lead I configuration). HRV was analyzed by applying a simple Fourier transform, obtaining a spectrum distribution curve of the frequency changes in the heart rate. The total power of HRV was calculated (TP), as well as the power in three bands: very low-frequency oscillations (HRV_VLF, 0–0.04 Hz), low-frequency oscillations (HRV_LF, 0.04–0.15 Hz) and high-frequency oscillations (HRV_HF, 0.15–0.4 Hz).

Beat-to-beat blood pressure was collected using the Penaz’s method with the finger cuff placed around the left middle finger. Similar to HRV, the total power of the spectrum of systolic (SBPV_TP, mmHg^2^) and diastolic (DBPV_TP, mmHg^2^) blood pressure and their frequency components were calculated.

The ultrasonic sensor of the SACR device allows to measure flows of air on inspiration and expiration and the respiration rate can be defined based on the respiration pattern. More detailed information on the methodology can be found in the study by Pivovarov [17].

Subjects were tested in a quiet room in the sitting position. Recordings were made during (a) spontaneous breathing (5 min); (b) controlled breathing at 0.2 Hz (i.e., 12 breaths/min for 2 min); and (c) controlled breathing at 0.1 Hz (i.e., 6 breaths/min for 2 min). All recordings were manually analyzed for the presence of artefacts.

Baroreflex sensitivity (BRS) was determined using the sequence method using artefact-free heart rate and blood pressure recordings during spontaneous breathing. The sequence method involves manual detection of up sequences, which are sequences with increasing SBP values which are followed by the corresponding lengthening of the R–R interval over three or more consecutive heart beats and down sequences (decreasing SBP values with a corresponding shortening of the R–R interval). BRS was calculated as the change in R–R interval (ms) over the change in SBP (mmHg). The baroreflex effectiveness index (BEI) was calculated as the ratio between the number of SBP ramps associated with corresponding changes in the R–R interval within two heart beats to the total number of SBP ramps [18].

### 2.3. Statistical Analysis

Statistical analysis was performed using the Statistica 10.0 software package (Software Inc., Atlanta, GA, USA). The results are shown as median (percentile 25–percentile 75). The differences between groups were assessed using the non-parametric Kruskal–Wallis test with a post hoc Dunn test using the PMCMRPlus R package [19]. The strength and significance of the correlation between selected variables were calculated using the non-parametric Spearman’s test. The multiple regression models and one-factor regression models, based on the HRV and RV parameters, were used in order to determine significant predictors for the diagnosis of ME/CFS. An ROC curve analysis was used to determine the appropriate cut-off values for the HRV and RV parameters. The level of significance for the Kruskal–Wallis test and correlation analysis was set at *p* < 0.05. In the post hoc Dunn’s test, the corrected α using the Bonferroni correction method was 0.01667.

## 3. Results

### 3.1. Demographic and Clinical Characteristics of Participants

Table 1 shows the descriptive statistics for the patients with ME/CFS, patients with PCC and participants in the control group. There were no differences between the groups in terms of age, gender or body mass index (BMI).

The ME/CFS and PCC patients experienced significant fatigue on all subscales of MFI-20, with obvious differences compared to the HCs. The patients had also higher scores of anxiety and depression (HADS) and lower levels of physical activity (total IPAQ score) compared with the HCs (Table 1). At the same time, the correlation analysis showed that depression and anxiety were associated with fatigue (general fatigue, physical fatigue, reduced activity and mental fatigue domains of MFI-20) only in the HCs (r values for the correlation between HADS-D/HADS-A and MFI-20 domains ranged from 0.373 to 0.612, *p* < 0.01) but not in the ME/CFS or PCC patients (*p* > 0.05). A total of 14/29 (48.3%) of patients with PCC in our study met the Canadian Consensus criteria of ME/CFS (2003) and 16/29 (55.2%) met the more recent IOM/NAM criteria (2015), which is consistent with the literature [3]. Physical activity levels (total score IPAQ) did not show any correlation with clinical characteristics in the ME/CFS or HC groups but correlated negatively with general fatigue (r = −0.404, *p* = 0.03) and physical fatigue (r = −0.486, *p* = 0.008) in the patients with PCC.

### 3.2. Baseline Variability Indices

The frequency-domain analysis of the RR intervals revealed significantly lower values for all analyzed HRV parameters (except for VLF band) in the PCC patients compared to the HCs (Table 2). Similar results were obtained for the group of ME/CFS patients, and the differences in HRV parameters between the ME/CFS and HC groups were even more pronounced. Additionally, the ME/CFS patients had lower values of VLF than the controls.

The spectral analysis of beat-to-beat arterial blood pressure revealed no differences between the ME/CFS or PCC patients and healthy controls in any of the frequency-domain parameters.

### 3.3. Variability Indices during Breathing at 12 Breaths per Minute

**Figure 1:**
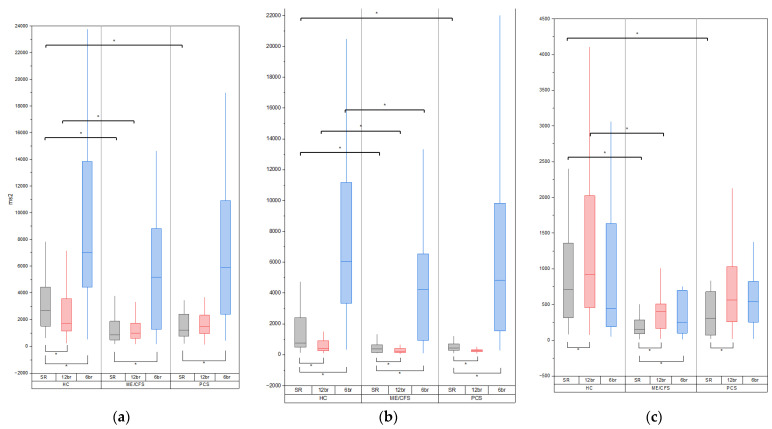
Heart rate variability (HRV) and respiration variability (RV) in three study groups (healthy controls (HCs) and myalgic encephalomyelitis/chronic fatigue syndrome (ME/CFS) and post-COVID-19 syndrome (PCS) patients) during spontaneous breathing (SR) at 12 breaths/min (12 br) and at 6 breaths/min (6 br). Significant differences in variability indices in ME/CFS and PCS patients compared to HCs and significant changes in these indices at 12 breaths/min and at 6 breaths/min within patient groups are marked with brackets and asterisks at the top and at the bottom of the plots respectively. We regarded *p* < 0.05 as statistically significant differences between the groups. In the post hoc Dunn’s test (which was performed to compare the data between groups), the corrected α using the Bonferroni correction method was 0.01667. (**a**) Total power (TP) of HRV (ms^2^); (**b**) low frequency (LF) HRV (ms^2^); (**c**) high frequency (HF) HRV (ms^2^).

During spontaneous breathing, the traditional spectral analysis showed decreased LF power and increased HF power of HRV at 12 breaths/min in all three groups (*p* < 0.05, see Figure 1a–c). The TP of HRV decreased only in the healthy controls. This decline in the HCs was mostly due to a decrease in VLF power, which could not be analyzed in short-term HRV recordings.

It should be noted that the patients with ME/CFS still had significantly lower TP, LF and HF values compared to the HCs. At the same time, the PCC patients did not differ from the HCs. There were some differences in SBPV and DBPV between the ME/CFS and HC groups (Table 3).

### 3.4. Variability Indices during Breathing at 6 Breaths per Minute

Compared spontaneous breathing, the traditional spectral analysis showed increased TP and LF power of the HRV at 6 breaths/min in all three groups (*p* < 0.05, see Figure 1a–c). The HF power of the HRV increased only in the ME/CFS and PCC groups.

At this respiration rate, the patients with ME/CFS still had significantly lower LF values compared to the HCs, but the TP and HF components were comparable. All HRV values of the PCC patients were comparable with those of the healthy controls. There were no differences in SBPV and DBPV between the groups (Table 3).

### 3.5. Multifactorial Logistic Regression Analysis of ME/CFS Group

A logistic regression was performed to ascertain if the HRV and RV parameters with a *p* value < 0.05 could predict the occurrence of ME/CFS. Two multifactorial models were created based on two different methods for variable selection. Model 1, which was based on forward stepwise logistic regression, included the TP of HRV and TP of RV during spontaneous breathing. Model 2, which was based on backward Wald selection, retained the LF of HRV and TP of RV during spontaneous breathing and the HF of HRV at 12 breaths/min (Table 4).

**Figure 2:**
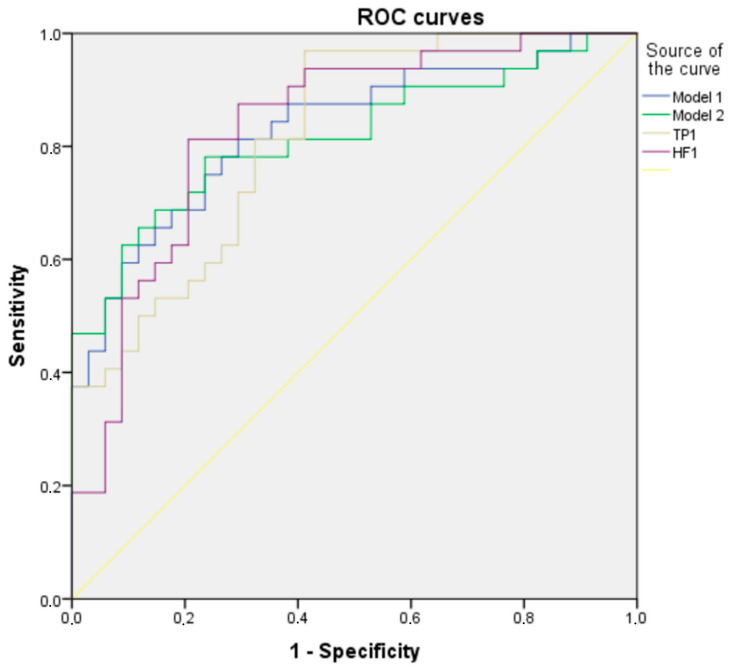
Receiver operating characteristic (ROC) curves for the analyzed models. TP1: total power of heart rate variability during spontaneous breathing; HF1: high frequency of heart rate variability during spontaneous breathing.

There was no collinearity between the parameters included in these models as the variance inflation factor (VIF) scores were well below 10. One-factor regression models, based on each significant (*p* value < 0.05) HRV and RV parameter, were also created. Two of these one-factor models had an AUCROC > 0.8 and were included in the comparison with multifactorial models (Table 5, Figure 2).

### 3.6. Correlations between Heart Rate and Arterial Blood Pressure Variability Parameters and Clinical Characteristics in Patient Groups

The Spearman correlation test was performed separately in the ME/CFS, PSC and HC groups to detect correlations between variability parameters and clinical characteristics (five domains of fatigue according to MFI-20; depression and anxiety according to HADS; age; BMI; physical activity according to IPAQ). The analysis showed considerably different patterns between the three groups (Table 6). The heart rate and arterial blood pressure variability parameters had a number of correlations with general fatigue and physical fatigue in the ME/CFS and PCC groups, but not in the healthy controls. In general, the HRV parameters had negative and SBPV/DBPV parameters had positive correlations with fatigue. Notably, fatigue showed many more correlations with the variability parameters than with depression. BMI correlated negatively with HRV, and these correlations were more pronounced in the patient groups and during slow breathing. The physical activity level (total score IPAQ) did not show any correlation with the variability parameters in any group.

### 3.7. Baroreflex Sensitivity and Baroreflex Effectiveness Index

The BRS was significantly lower both for up and down SBP ramps in the patients with ME/CFS compared to the HCs and significantly lower only for down SBP ramps in the patients with PCC (Table 7). Interestingly, BEI was reduced only in up SBP ramps in both groups of patients but it did not reach statistical significance, although the number of ineffective baroreflex events in the up sequences of SBP was significantly higher than in the HCs.

The Spearman correlation test was performed separately in the ME/CFS, PSC and HC groups to detect correlations between BRS, BEI_up and clinical characteristics (five domains of fatigue according to MFI-20; depression and anxiety according to HADS; age; BMI; physical activity according to IPAQ). In the HCs, BRSup correlated negatively with age (−0.406) and BEI_up correlated negatively with the activity domain of fatigue according to MFI-20 (−0.416), but not with the level of physical activity according to IPAQ. Only in the ME/CFS only group the fatigue domains showed significant correlation with BRSup, BRSdown, BRSmean (−0.559, −0.394 and −0.546, respectively, with general fatigue domain of MFI-20) and BEI_up (−0.404 with motivation domain of fatigue in MFI-20). In the PCC patients, no significant relationships were found.

## 4. Discussion

This study assessed autonomic function at rest, during breathing at 12 breaths per minute and during breathing at 6 breaths per minute in patients with PCC and in patients with ME/CFS whose disease was not related to COVID-19. A correlation analysis of HRV and BPV indices with clinical features of the patients was performed to better understand the determinants of autonomic dysfunction or their contribution to the clinical presentation. The obtained HRV indices were used to develop a multifactorial model for predicting the likelihood of an ME/CFS diagnosis. The assessment of baroreflex function could enable deeper insights into the pathogenesis of orthostatic intolerance in both PCC and ME/CFS.

### 4.1. Clinical Characteristics of the Patients with ME/CFS and PCC

First of all, it should be noted that the study groups were matched by age, gender and BMI to eliminate the influence of these factors on HRV and baroreflex function. All patients and controls were under 60 years old, which enable us to ignore the effects of aging on the autonomic nervous system. The patients with PCC and ME/CFS were significantly more anxious and depressed than the HCs according to HADS; however, the median level of both anxiety and depression in the patient groups corresponded to the subclinical level (i.e., did not warrant treatment but may nevertheless interfere with an individual’s ability to function effectively). At the same time, the MFI-20 subscale scores in both patient groups were similar and corresponded to severe fatigue. The patients were less physically active compared to the HCs according to their IPAQ scores. In our study, the patients with PCC were comparable with ME/CFS patients and were more physically active than patients with PCC in the study of Twomey et al. [20] (the authors reported a total IPAQ score of 503 (99-1361) MET-min/week for patients with long COVID).

### 4.2. HRV and BPV in Patients with ME/CFS and PCC

The absolute values of LF and HF HRV in the ME/CFS and PCC patients were lower than in the healthy subjects. This observation could indicate reduced sympathetic and parasympathetic activity in the ME/CFS and PCC patients at rest. There are serious doubts about the assessment of cardiac autonomic nervous functions through the analysis of HRV frequency components (LF and HF) [21]. In particular, the framework to associate LF and HF with the divisions of the autonomic nervous system (sympathetic and parasympathetic) is already too simplistic. It is known that two major components in short-term HRV are respiratory sinus arrhythmia (RSA) and a fluctuation at ~0.1 Hz (which corresponds to LF band) called a Mayer wave sinus arrhythmia. The mechanism mediating the heart rate fluctuation at ~0.1 Hz is considered to be due to the baroreceptor reflex cardiovascular regulation system, which causes the reflection of the fluctuation of arterial blood pressure at ~0.1 Hz that is known as Mayer waves in the fluctuation of the heart rate [21]. As LF is related to baroreflex function, a decreased LF HRV, which is constantly reported in ME/CFS, could reflect a baroreflex failure and encouraged us to perform beat-to-beat measures of HR and BP in order to confirm this hypothesis.

Regarding the second mechanism of short-term HRV, respiration modulates the heart rate, creating excess power in the HRV at a frequency equal to the respiratory rate, a phenomenon known as RSA, which has been associated with the efficiency of pulmonary gas exchange [22]. RSA is generated by the oscillation of firing in cardiac vagal motor neurons, which are inhibited during inspiration and excited after inspiration [23]. Natural breathing rates between 10 and 24 cycles per minute (0.16–0.4 Hz) allows us to estimate parasympathetic activity through measuring HF HRV power, since RSA at this breathing rate corresponds to the HRV HF band and does not overlap with the LF HRV band.

Our results for the comparison of HRV indices between spontaneous breathing rate and during breathing at 12 breaths per minute (i.e., the increase in HF power and decrease in LF power in all three groups) are in line with the separation of the two major sources of HRV (RSA and Mayer wave sinus arrhythmia) by frequency bands at this respiratory rate. Therefore, the decreased LF and HF HRV in the ME/CFS group compared to the HCs at 12 breaths per minute confirmed a reduced parasympathetic activity and give additional evidence for a baroreflex failure in both patient groups.

It is very important to take into consideration individuals’ breathing rate when HF and LF components of HRV are interpreted as indicators of parasympathetic and sympathetic nervous system activity. Thus, for athletes, who breathe more slowly (for example at 6 cycles per minute, which corresponds to a frequency of 0.1 Hz), vagus-related power would shift from HF into LF [24], resulting in an overestimation of the SNS contribution and underestimation of the PNS contribution in the traditional HRV framework. The exact mechanism of the LF increase in this case is not through an increase in sympathetic activity, but the synchronization of RSA with the baroreflex frequency [24].

The maximization of HRV at around 6 breaths per min due to LF power has been confirmed by numerous studies [22]. This indicates cardiorespiratory system resonance, and slow breathing (~6 breaths per min) is hence referred to as a “resonance breathing”, although this resonant frequency does vary between individuals (4–7.5 breaths per min) [22]. Slow breathing has been shown to benefit health and performance within clinical and non-clinical populations. EEG studies highlighted an increase in alpha and a decrease in theta power. An FMRI study reported increased activity in cortical (e.g., prefrontal, motor and parietal cortices) and subcortical (e.g., pons, thalamus, sub-parabrachial nucleus, periaqueductal gray and hypothalamus) structures. The psychological/behavioral outputs related to the abovementioned changes included reduced symptoms of arousal, anxiety, depression, anger and confusion as well as increased comfort, relaxation, pleasantness, vigor and alertness [25].

In our study, the effect of slow breathing (6 breaths/min) on HRV in the patients and HCs was consistent with earlier studies [22], which implies the integrity of the positive effects of slow breathing (and in particular resonance frequency breathing) in ME/CFS and PCC. Moreover, our results suggest that slow breathing has a therapeutic potential regarding PCC since the HRV parameters in this group at 6 breaths/min increased to the same level as the HCs. Autonomic dysfunction in ME/CFS is presumably more profound than in PCC, since in the ME/CFS patients, it was not resolved during breathing at 12 and 6 breaths/min.

In view of the observed great difference in HRV parameters between the ME/CFS and HC groups as well as the absence of a clinically available diagnostic test for ME/CFS, we used a multifactorial logistic regression analysis to create two multifactorial models for the prediction of ME/CFS based on HRV parameters. However, two single-factor models based on TP of HRV and HF of HRV during spontaneous breathing showed an AUC, sensitivity and specificity comparable with multifactorial models. The most optimal model, in our view, was based on HF HRV during spontaneous breathing and showed a sensitivity of 81.3% and specificity of 79.4% for the differentiation of ME/CFS patients from HCs (with a cut-off of 286.95 ms^2^).

### 4.3. Correlation Analysis of HRV and BPV Indices with Clinical Features of the Patients

The correlation analysis revealed some important differences between the patients and HCs. First of all, depression and anxiety were associated with fatigue only in the HCs, but not in the ME/CFS or PCC patients. The association of fatigue with depression is well known and a differential diagnosis of ME/CFS or PCC with depression is often difficult due to the overlapping psychological and somatic symptoms in both disorders, including fatigue, reduced concentration, and sleep disturbances. Our study showed that fatigue was not related to depression/anxiety in ME/CFS and PCC, which is highly interesting in view of the renewed attempts of a psychologization of ME/CFS and PCC and represents a strong argument against this concept.

Depression was not associated with HRV parameters in any study groups, while anxiety was inversely correlated with HRV-HF during spontaneous breathing in the HCs. Since, as have been mentioned above, low HRV-HF was characteristic of ME/CFS and PCC, one may presume that low HRV-HF in the HCs with subclinical anxiety represents a risk factor for post-infectious fatigue syndromes. Correlations of “general fatigue” and “physical fatigue” domains of MFI20 were the most indicative for the hypothesis that fatigue in ME/CFS and PCC is related to the autonomic dysfunction in contrast to the HCs, where depression/anxiety are the most common causes of fatigue. In general, lower HRV and higher SBPV/DBPV in the patients were associated with more pronounced fatigue. These results are in line with previous studies which have been scarce, but reported similar relationships [26,27,28]. In general, our data provide additional evidence that fatigue is not the consequence of depression in ME/CFS and PCS but related to dysautonomia. The association of fatigue with depression is a popular psychological concept and it was even supported in our study in the case of the HCs but not in case of the ME/CFS and PCS patients.

Interestingly, in our study, some HRV and SBPV parameters (which were associated with greater fatigue only in the patient groups), both in patients and HC, were correlated with age, while age itself was not correlated with any domain of fatigue. These findings could be interpreted as evidence for the existence of age-related autonomic dysfunction, which is accelerated in ME/CFS and PCC. A similar pattern was observed for another measure of autonomic cardiovascular control—the baroreceptor reflex.

### 4.4. Baroreflex Sensitivity in Patients

BRS reflects the complex interaction between the cardiac, vascular and autonomic systems that maintain the blood pressure fluctuations of daily life within normal levels. The afferents arising from the arterial baroreceptors terminate in the nucleus tractus solitarii of the brainstem, which activates vagal motor neurons and inhibits spinal sympathetic neurons. When the BP rises, the baroreflex stimulates vagal cardiac activity (increasing HRV), inhibits β1-adrenergic efferents to the myocardium (reducing contractility), and reduces α-adrenergic outflow to the vasculature (causing vasodilation). Conversely, when the BP decreases, the opposite changes occur, such that the reflex buffers short-term BP fluctuations.

A close relationship between orthostatic intolerance, fatigue, cognitive impairment, chronic pain and baroreflex failure has been recently reported [29,30].

Our results are in line with these observations, since a baroreflex failure was detected in both ME/CFS and PCC patients. However, in the patients with PCC, baroreflex failure was not correlated with fatigue in contrast to the ME/CFS patients. It should be noted that there are conflicting data on the presence of baroreflex failure in PCC in the literature [31]. The negative correlation of BRSup with age in the HCs (but not in the ME/CFS patients where it was correlated with the severity of fatigue) provides more evidence for the hypothesis of the accelerated aging of the autonomic nervous system in ME/CFS.

The decreased number of the effective baroreflex events in the ME/CFS patients in only up SBP ramps could represent a new mechanism for the higher risk of POTS and inappropriate sinus tachycardia in this group of patients.

Several potential mechanisms connecting an abnormal baroreflex to fatigue and cognitive impairment has been suggested, including reduced or dysregulated cerebral perfusion, the effects of decreased vagal afferent inputs to the nucleus tractus solitarii on higher cortical networks [30], and deactivation of the physiological baroreflex-induced reduction in systemic inflammation and neuroinflammation [32,33]. Therefore, baroreflex dysfunction possibly contributes to the reduction in cerebral blood flow, which has been reported in ME/CFS and PCC [5]. In our opinion, both dysautonomia and decreased sensitivity of the arterial baroreceptors could be the basis for the baroreflex dysfunction in ME/CFS and PCC.

### 4.5. Mechanisms of Dysautonomia in ME/CFS and PCC and a Potential Link with Cardiovascular Diseases

The cause of the dysautonomia in ME/CFS and PCC is not entirely clear. Deconditioning was initially thought to be a mechanism for the development of the associated dysautonomia but other reports have argued against it [34]. We also did not observe any relationship between the level of physical activity and dysautonomia in the ME/CFS or PCC patients. Other mechanisms of dysautonomia in these conditions described in the literature are direct neuronal injury of the autonomic pathways (e.g., small fiber neuropathy) and indirect immune-mediated disturbances (e.g., the effects of anti-GPCR autoantibodies) [35,36]. The change in autonomic nervous system activity against the background of neuroinflammation (which was reported in both ME/CFS [4] and PCC [37]) may be a potential intermediate link between microglia and cardiovascular diseases [38]. The ANS innervates vascular walls and regulates contractility and tension. Therefore, autonomic dysfunction may exert detrimental effects on endothelial and vascular tissues, favoring atherogenesis. A relationship between dysautonomia and atherosclerotic cardiovascular disease mortality has been documented [39]. Subclinical atherosclerosis risk factors and markers were associated with signs of dysautonomia in general populations with various ethnic origins [40,41]. Sympathetic hyper-activation leads to vasoconstriction, the loss of vascular elasticity and the accumulation of modified lipoproteins in the vascular wall. It also increases peripheral vascular resistance, induces endothelial dysfunction, stimulates oxidative stress and vascular remodeling, and favors micro- and macro-calcification in both the vascular intima and media [42,43]. Sympathetic hyper-activation also favors pro-inflammatory and prothrombotic effects [42] driven by cytokines, chemokines and other biologically active mediators, while the parasympathetic branch of the autonomic nervous system plays an anti-inflammatory role mediated by the so-called cholinergic anti-inflammatory pathway. This pathway is characterized by signals communicated via the vagus nerve (with the possible involvement of the splenic nerves) through acetylcholine release to down-regulate the inflammatory actions of macrophages, which are key players in inflammaging [44]. The hypothalamic–pituitary–adrenal axis is another pathway related to the ANS, which promotes some anti-inflammatory responses mainly through increased cortisol levels (notably, this mechanism is also impaired in ME/CFS [45]). In line with these pathophysiological mechanisms, reduced HRV (which we observed in the ME/CFS and PCC patients in this study) predicted the severity, extent and progression of human coronary atherosclerosis, even in asymptomatic subjects [46,47].

## 5. Limitations and Conclusions

There are limitations to the present study. We assessed the group of PCC patients as a whole. But, in our study, this group was heterogenous (some of the patients met the ME/CFS diagnostic criteria and some of them did not). It would be interesting to examine these subgroups of PCC separately. Further, HRV and BPV were assessed only once; therefore, we cannot claim the stability of the results. In future studies, a longitudinal assessment of patients should be used to clarify whether the HRV and BPV parameters in ME/CFS and PCC are stable or ameliorated over time or with treatment.

In conclusion, we obtained evidence for similar patterns of HRV, blood pressure variability and baroreflex failure in ME/CFS and PCC patients, which can be interpreted as a pathological acceleration of age-dependent dysautonomia and applied for the diagnosis of ME/CFS. Our results are in line with the observations of other authors that the symptomatology and objective signs of dysautonomia are comparable in patients with PCC and in patients with ME/CFS triggered with Epstein–Barr virus and with an insidious onset [5]. These findings suggest that PCC and ME/CFS are probably the same disease which can be caused by different triggers. The correlation analysis revealed a close relationship between fatigue and parameters which objectively prove dysautonomia in ME/CFS and PCC, but not with depression/anxiety. We discussed potential mechanisms of dysautonomia in ME/CFS and PCC and drew attention to the potential negative vascular consequences of these conditions.

## Figures and Tables

**Table 1:** Baseline demographic and clinical parameters of participants.

Variable	ME/CFS	PCC	HC	*p*-Value of Kruskal– Wallis Test	*p*-Value of Dunn Test ME/CFS vs. HC	*p*-Value of Dunn Test PCC vs. HC
Age (years)	35.00 (30.0–41.25)	35.00 (29.50–41.50)	34.50 (21.25–43.75)	0.302	N/A	N/A
BMI (kg/m^2^)	23.41 (19.45–28.70)	23.51 (20.54–27.86)	22.07 (20.14–25.41)	0.649	N/A	N/A
	Gender					
Male	8 (23.53%)	6 (20.69%)	10 (31.25%)	0.612 *	N/A	N/A
Female	26 (76.47%)	23 (79.31%)	22 (68.75%)			
	HADS					
Anxiety	10.00 (5.75–12.00)	10.00 (6.00–12.00)	6.00 (2.00–8.00)	0.000	0.001	0.001
Depression	10.00 (7.75–11.25)	9.00 (6.00–10.00)	3.00 (1.25–4.00)	0.000	0.000	0.000
	MFI-20					
General fatigue	19.00 (17.75–20.00)	18.00 (14.50–20.00)	8.00 (5.25–9.75)	0.000	0.000	0.000
Physical fatigue	17.50 (15.00–19.00)	16.00 (14.00–18.00)	7.00 (5.00–9.75)	0.000	0.000	0.000
Reduced activity	18.00 (16.00–19.00)	16.00 (12.5–19.00)	7.50 (4.25–11.00)	0.000	0.000	0.000
Reduced motivation	13.50 (11.00–16.00)	12.00 (9.50–15.00)	8.00 (6.00–9.00)	0.000	0.000	0.000
Mental fatigue	16.50 (15.00–18.00)	13.00 (11.00–16.00)	6.00 (4.00–9.00)	0.000	0.000	0.000
	IPAQ					
Total score IPAQ (MET-min/Week)	1857.00 (590.25–2841.00)	1506.00 (1039.50–2814.00)	3027.00 (1606.50–5399.00)	0.007	0.004	0.010

Medians [IQR: interquartile ranges] and percentages (%) are shown. BMI: body mass index; HADS: Hospital Anxiety and Depression Scale; HC: healthy control; IPAQ: International Physical Activity Questionnaires; ME/CFS: myalgic encephalomyelitis/chronic fatigue syndrome; MFI: Multidimensional Fatigue Inventory; PCC: post-COVID-19 condition. In Kruskal–Wallis tests, *p* < 0.05 was regarded as indicating statistically significant differences between two groups. In Post hoc Dunn’s tests, the corrected α using the Bonferroni correction method was 0.01667. * Chi square test was performed for “Gender”.

**Table 2:** Baseline parameters of heart rate variability among participants.

Variable	ME/CFS	PCC	HC	*p*-Value of Kruskal– Wallis Test	*p*-Value of Dunn Test ME/CFS vs. HC	*p*-Value of Dunn Test PCC vs. HC
	Heart rate variability					
TP (ms^2^)	852.45 (469.03–1912.88)	1358.10 (834.15–2687.15)	2709.05 (1483.38–4454.36)	0.000	0.000	0.003
LF (ms^2^)	367.10 (137.45–635.13)	429.20 (279.65–867.70)	759.30 (477.33–2480.68)	0.000	0.000	0.013
HF (ms^2^)	152.90 (91.93–284.78)	335.00 (102.45–717.10)	703.70 (311.00–1394.80)	0.000	0.000	0.006
VLF (ms^2^)	256.15 (192.03–619.38)	469.20 (237.10–814.60)	727.50 (431.85–1014.80)	0.000	0.000	0.027
LF/HF	2.14 (1.20–4.13)	1.46 (1.02–4.01)	1.25 (0.71–2.31)	0.080	N/A	N/A
	Beat-to-beat systolic arterial blood pressure variability					
TP (mmHg^2^)	50.15 (23.38–66.78)	42.20 (26.85–65.80)	41.35 (22.15–63.15)	0.763	N/A	N/A
LF (mmHg^2^)	13.95 (6.45–21.85)	13.50 (8.80–23.30)	9.50 (6.30–19.68)	0.550	N/A	N/A
HF (mmHg^2^ms^2^)	7.80 (4.38–16.73)	8.40 (3.60–11.45)	8.50 (4.48–14.28)	0.987	N/A	N/A
VLF (mmHg^2^ms^2^)	20.30 (10.08–31.83)	17.50 (7.80–29.60)	12.90 (5.63–33.60)	0.388	N/A	N/A
LF/HF	1.63 (0.77–2.43)	1.79 (1.20–2.92)	1.11 (0.65–2.85)	0.314	N/A	N/A
	Beat-to-beat diastolic arterial blood pressure variability					
TP (mmHg^2^ms^2^)	12.95 (7.38–25.28)	12.80 (8.05–25.35)	11.75 (7.60–20.53)	0.738	N/A	N/A
LF (mmHg^2^ms^2^)	5.15 (2.93–7.98)	5.30 (3.15–8.70)	4.45 (2.25–7.90)	0.882	N/A	N/A
HF (mmHg^2^ms^2^)	1.50 (0.90–2.15)	1.10 (0.70–2.50)	1.45 (0.70–2.68)	0.945	N/A	N/A
VLF (mmHg^2^ms^2^)	5.75 (3.03–12.15)	6.20 (2.80–13.15)	4.45 (2.83–13.48)	0.882	N/A	N/A
LF/HF	3.39 (1.84–5.53)	3.91 (2.35–6.83)	3.02 (2.17–5.74)	0.885	N/A	N/A

Medians [IQR: interquartile ranges] are shown. HC: healthy control; HF: high frequency; ME/CFS: myalgic encephalomyelitis/chronic fatigue syndrome; LF: low frequency; PCC: post-COVID-19 syndrome; TP: total power; VLF: very low frequency. In Kruskal–Wallis tests, *p* < 0.05 was regarded as indicating statistically significant differences between two groups. In post hoc Dunn’s tests, the corrected α using the Bonferroni correction method was 0.01667.

**Table 3:** Heart rate and arterial blood pressure variability of participants at 12 breaths/min and at 6 breaths/min respiration rates.

Variable	ME/CFS	PCC	HC	*p*-Value of Kruskal–Wallis Test	*p*-Value of Dunn Test ME/CFS vs. HC	*p*-Value of Dunn Test PCC vs. HC
	Heart rate variability at 12 breaths/min					
TP (ms^2^)	998.90 (573.93–1729.15)	1506.80 (948.90–2410.35)	1682.35 (1120.95–3607.90)	0.002	0.000	0.108
LF (ms^2^)	226.55 (140.28–399.68)	272.60 (198.90–329.50)	402.7 (237.35–908.75)	0.005	0.002	0.023
HF (ms^2^)	399.15 (162.38–529.75)	564.50 (253.85–1095.35)	920.45 (439.75–2023.70)	0.001	0.0021	0.050
	Beat-to-beat systolic arterial blood pressure variability at 12 breaths/min					
TP (mmHg^2^ms^2^)	49.30 (24.85–65.45)	42.40 (26.00–97.60)	33.00 (18.85–49.18)	0.223	N/A	N/A
LF (mmHg^2^)	11.90 (5.93–21.33)	9.60 (4.75–23.10)	5.75 (3.70–10.85)	0.018	0.009	0.023
HF (mmHg^2^ms^2^)	14.74 (6.08–32.05)	12.80 (6.40–35.80)	13.80 (8.38–22.50)	0.972	N/A	N/A
	Beat-to-beat diastolic arterial blood pressure variability at 12 breaths/min					
TP (mmHg^2^ms^2^)	12.35 (7.48–22.78)	13.90 (7.15–20.60)	9.35 (4.63–14.55)	0.071	N/A	N/A
LF (mmHg^2^ms^2^)	4.10 (2.13–6.90)	4.10 (1.85–8.15)	2.70 (1.73–4.70)	0.158	N/A	N/A
HF (mmHg^2^)	3.35 (1.45–5.83)	2.60 (1.00–6.40)	1.45 (0.93–3.15)	0.041	0.013	0.095
	Heart rate variability at 6 breaths/min					
TP (ms^2^)	5179.75 (1260.90–8857.20)	5891.10 (2363.10–11,520.45)	7007.30 (4443.65–14,608.75)	0.061	N/A	N/A
LF (ms^2^)	4220.15 (921.70–6590.25)	4824.30 (1528.40–9957.70)	6022.40 (3333.65–11,796.05)	0.034	0.009	0.126
HF (ms^2^)	249.90 (98.28–698.98)	536.10 (231.55–1099.25)	442.90 (183.50–1643.08)	0.093	N/A	N/A
	Beat-to-beat systolic arterial blood pressure variability at 6 breaths/min					
TP (mmHg^2^ms^2^)	77.20 (43.58–136.63)	84.40 (48.90–157.35)	62.90 (39.38–96.05)	0.258	N/A	N/A
LF (mmHg^2^ms^2^)	53.60 (22.05–106.18)	57.70 (27.30–122.60)	39.25 (22.23–69.70)	0.166	N/A	N/A
HF (mmHg^2^ms^2^)	5.35 (2.80–10.85)	5.20 (2.65–8.95)	3.15 (1.93–6.23)	0.058	N/A	N/A
	Beat-to-beat diastolic arterial blood pressure variability at 6 breaths/min					
TP (mmHg^2^ms^2^)	21.15 (11.45–39.48)	17.90 (12.70–35.20)	15.30 (10.45–27.23)	0.525	N/A	N/A
LF (mmHg^2^ms^2^)	14.25 (5.23–26.93)	12.60 (6.35–27.00)	9.40 (5.05–20.80)	0.463	N/A	N/A
HF (mmHg^2^ms^2^)	1.70 (1.10–2.53)	2.10 (1.40–3.80)	1.10 (0.70–3.38)	0.142	N/A	N/A

Medians [IQR: interquartile ranges] are shown. HC: healthy control; HF: high frequency; ME/CFS: myalgic encephalomyelitis/chronic fatigue syndrome; LF: low frequency; PCC: post-COVID-19 condition; TP: total power. In Kruskal–Wallis tests, *p* < 0.05 was regarded as indicating statistically significant differences between two groups. In post hoc Dunn’s tests, the corrected α using the Bonferroni correction method was 0.01667.

**Table 4:** Multivariate logistic regression analysis for predicting the presence of ME/CFS.

Variable	Adj. B	Adj. OR (95% CI)	*p* Value
Model 1			
TP of HRV during spontaneous breathing	−0.001	0.999 (0.999–1.002)	0.001 *
TP of RV during spontaneous breathing	0.002	1.002 (1.000–1.004)	0.044 *
Hosmer Lemeshow test, *p*-value = 0.952; constant = 0.504			
Model 2			
LF of HRV during spontaneous breathing	−0.001	0.999 (0.998–1.000)	0.012 *
HF of HRV at 12 breaths/min	−0.001	0.999 (0.998–1.000)	0.047 *
TP of RV during spontaneous breathing	0.002	1.002 (1.000–1.003)	0.088
Hosmer Lemeshow test, *p*-value = 0.730; constant = 0.562			

RV: respiration variability; HF: high frequency; HRV: heart rate variability; LF: low frequency; TP: total power; VLF: very low frequency. We regarded * *p* < 0.05 as indicating statistically significant differences between two groups.

**Table 5:** Comparison of predictive performance in receiver operating characteristic analysis.

Variable	TP of HRV at SR	HF of HRV at SR	Model 1	Model 2
AUC (95% CI)	0.819 (0.720–0.918)	0.834 (0.735–0.933)	0.830 (0.730–0.929)	0.817 (0.712–0.922)
Cut-off (maximum Youden’s index)	1047.95	286.95	0.580	0.496
Sensitivity %, (95% CI)	96.9%	81.3%	73.5%	85.3%
Specificity %, (95% CI)	58.8%	79.4%	75%	68.8%
Cut-off (Se = Sp)	1587.55	296.95	0.603	0.612
Sensitivity %, (95% CI)	71.9%	78.1%	70.6%	76.5%
Specificity %, (95% CI)	70.6%	79.4%	81.3%	75%

AUC: area under the curve; CI: confidence interval; HF: high frequency; HRV: heart rate variability; SR: spontaneous breathing (rest); TP: total power.

**Table 6:** Spearman’s rank correlation coefficients (*p* < 0.01) of heart rate and arterial blood pressure variability parameters with the clinical characteristics of participants in each study group.

	Age	BMI	HADS-D	HADS-A	GF	PF	RA	RM	MF	Total IPAQ Score
**Spontaneous respiration**										
HC										
− *										
ME/CFS										
LF_HR_		−0.462								
PCC										
LF_HR_	−0.477									
**Breathing at 12 breaths per minute**										
HC										
− *										
ME/CFS										
HF_SBP_		−0.442								
TP_HR_					−0.444					
HF_HR_					−0.522					
PCC										
HF_DBP_	−0.497									
LF_SBP_						0.492				
**Breathing at 6 breaths per minute**										
HC										
LF_SBP_	0.465									
ME/CFS										
TP_SBP_	0.441									
LF_SBP_	0.452					0.443				
TP_HR_		−0.452								
LF_HR_		−0.486								
TP_DBP_						0.513				
LF_DBP_						0.601				
PCC										
TP_HR_		−0.523								
LF_HR_		−0.547								
HF_HR_		−0.584								

* no correlations with *p* < 0.01 were identified. BMI: body mass index; DBP: diastolic blood pressure; GF: general fatigue, MFI-20 domain; HADS_A: Hospital Anxiety and Depression Scale—Anxiety subscale; HADS_D: Hospital Anxiety and Depression Scale—Depression subscale; HC: healthy control; HF: high frequency; HRV: heart rate variability; IPAQ: International Physical Activity Questionnaires; LF: low frequency; ME/CFS: myalgic encephalomyelitis/chronic fatigue syndrome; MF: mental fatigue, MFI-20 domain; PCC: post-COVID-19 syndrome; PF: physical fatigue MFI-20 domain; RA: reduced anxiety, MFI-20 domain; RM: reduced motivation, MFI-20 domain; SBP: systolic blood pressure; TP: total power; VLF: very low frequency.

**Table 7:** Baroreflex sensitivity and baroreflex effectiveness index of participants.

Variable	ME/CFS	PCC	HC		*p*-Value ME/CFS vs. HC	*p*-Value PCC vs. HC
BRSup	4.42 (2.88–6.28)	5.91 (3.54–7.92)	7.40 (4.90–14.03)	0.001	0.000	0.065
BRSdown	4.85 (2.93–7.42)	5.24 (3.97–8.48)	9.15 (6.42–12.01)	0.000	0.000	0.003
BRSmean	4.60 (3.12–6.40)	5.99 (3.88–8.48)	8.45 (5.25–13.40)	0.001	0.000	0.024
BEI_up	0.57 (0.43–0.80)	0.64 (0.44–0.78)	0.70 (0.56–0.88)	0.045	0.020	0.064
BEI_down	0.45 (0.37–0.62)	0.44 (0.29–0.77)	0.49 (0.36–0.82)	0.680	N/A	N/A
BR_up	16.00 (7.50–25.00)	18.00 (10.00–27.50)	18.50 (9.25–29.00)	0.597	N/A	N/A
BR_down	19.00 (9.00–25.75)	12.00 (6.00–24.00)	19.5 (7.00–32.75)	0.346	N/A	N/A
BRX_up	10.5 (4.75–18.00)	9.00 (5.50–14.00)	5.50 (2.00–9.00)	0.014	0.007	0.023
BRX_down	18.5 (9.00–25.00)	14.00 (5.00–23.00)	16.00 (5.00–21.50)	0.338	N/A	N/A

BMI: body mass index; BEI: baroreflex effectiveness index; BR: number of effective baroreflex events; BRX: number of ineffective baroreflex events; BRS: baroreflex sensitivity; HC: healthy control; ME/CFS: myalgic encephalomyelitis/chronic fatigue syndrome; PCC: post-COVID-19 condition. In Kruskal–Wallis tests, *p* < 0.05 was regarded as indicating statistically significant differences between two groups. In the post hoc Dunn’s tests, the corrected α using the Bonferroni correction method was 0.01667.

## Data Availability

Data are contained within the article.
